# Supplementary material for: Modeling di (2-ethylhexyl) Phthalate (DEHP) and Its Metabolism in a Body’s Organs and Tissues through Different Intake Pathways into Human Body
Source: Int J Environ Res Public Health. 2022 May 9;19(9):5742. doi: 10.3390/ijerph19095742 (PMC9101911; doi:10.3390/ijerph19095742)
Supplement: Supplementary file 1 [file ijerph-19-05742-s001.zip › ijerph-1701639-supplementary.pdf]

Modeling DEHP and its metabolism in a body's organs and tissues through different intake pathways into human body

*Ao Li[1], Lingyi Kang[1], Runjie Li[1], Sijing Wu[1], Ke Liu[1], Xinke Wang[1]**

1.School of Human Settlements and Civil Engineering, Xi'an Jiaotong University, 710049, Xi'an, China

* Correspondence: wangxinke@mail.xjtu.edu.cn

Supporting Materials: Standard ordinary differential equations used in tissue dosimetry model

The equations are set to describe the scaling method used to the whole PBPK model and the governing equations in each organ or tissue can be found in Supporting Information.

The governing equation of intestine is shown as follows:

$$\frac{d(Agut)}{dt} = Qgut * \left(cplasma * fu - cgut * \frac{fu}{k\_gut\_plasma}\right) - RAMG1 - RAMG1\_cyt \quad 1$$

where $Agut$ is the amount of DEHP in gut; $Qgut$ is the blood flow to the gut; $cgut$ is the concentration of chemical in gut; fu is the plasma fractional unbound for DEHP; k_gut_plasma is the gut plasma partition coefficient; $RAMG1$ is metabolism of DEHP into MEHP in microsomal fraction of gut; $RAMG1\_cyt$ is metabolism of DEHP into MEHP in cytosol fraction of gut.

The equation2 describes the governing equation in liver.

$$\frac{d(Aliver)}{dt} = Qliver * cplasma * fu + \left(Qgut * cgut * \frac{fu}{k\_liver\_plasma}\right) - (Qliver + Qgut) * cliver * \frac{fu}{k\_liver\_plasma} - RAML1 - RAML1\_cyt \quad 2$$

where $Aliver$ is the amount of DEHP in liver; $Qliver$ is the cardiac blood flow to liver; $cplasma$ is the plasma concentration of DEHP; $cliver$ is the concentration of DEHP in liver; k_liver_plasma is the liver plasma partition coefficient.

The control equation of adipose tissue is shown as follows.

$$\frac{d(Afat)}{dt} = Qfat * \left(cplasma * fu - cfat * \frac{fu}{k\_fat\_plasma}\right) \quad 3$$

where Afat is the amount of chemical in fat; Qfat is the blood flow to fat; k_fat_plasma is the fat plasma partition coefficient.

The control equation to simplify the rest of the body is shown as follows.

$$\frac{d(Arestbody)}{dt} = Qrestbody * \left(cplasma * fu - crestbody * \frac{fu}{k\_restbody\_plasma}\right) \quad 4$$

where Arestbody is the amount of chemical in rest of the body; Qrestbody is the blood flow to rest of the body; k_restbody_plasma is the rest of the body plasma partition coefficient.

The equation5 is the governing equation described the metabolite rate in plasma(both venous blood and arterial blood).

$$\frac{d(Aplasma)}{dt} = Qfat * cfat * \frac{fu}{k\_fat\_plasma} + \left((Qliver + Qgut) * cliver * \frac{fu}{k\_liver\_plasma}\right)$$
$$+ Qrestbody * crestbody * \frac{fu}{k\_restbody\_plasma} - QCplasma * cplasma * fu \quad 5$$

where $Aplasma$ is the amount of chemical in plasma; $Qplasma$ is the blood flow to plasma; $QCplasma$ is the the cardiac output for plasma flow.

Other standard ordinary differential equations are shown as follows.

$$\frac{d(AgutM1)}{dt} = -kgut * AgutM1 + RAMG1 + RAMG1\_cyt - RAMG2 - RAMG5 \quad 6$$

$$\frac{d(AgutM2)}{dt} = RAMG2 - kgutM2 * AgutM2 - RAMG4 \quad 7$$

$$\frac{d(ALiverM1)}{dt} = Qgut * AgutM1 + Qliver * \left(cplasmaM1 * fum - cliverM1 * \frac{fum}{k\_liver\_plasma}\right)$$
$$+ RAML1 + RAML1\_cyt - RAML2 - RAML3 - RAML5 \quad 8$$

$$\frac{d(AliverM2)}{dt} = RAML2 + kgutM2 * AgutM2 - fracl2 * AliverM2 - RAML4 \quad 9$$

$$\frac{d(AfatM1)}{dt} = Qfat * \left(cplasmaM1 * fum - cfatM1 * \frac{fum}{k\_fat\_plasma}\right) \quad 10$$

$$\frac{d(ArestbodyM1)}{dt} = Qrestbody * \left(cplasmaM1 * fum - crestbodyM1 * \frac{fum}{k\_restbody\_plasmaM1}\right) \quad 11$$

$$\frac{d(AplasmaM1)}{dt} = Qfat * cfatM1 * \frac{fum}{k\_liver\_plasmaM1} + Qliver * cliverM1 * \frac{fum}{k\_liver\_plasmaM1}$$
$$+ Qrestbody * crestbodyM1 * \frac{fum}{k\_restbody\_plasmaM1} - QCplasma * cplasmaM1 * fum - kurineM1 * AplasmaM1 \quad 12$$

$$\frac{d(AurineM1)}{dt} = kurineM1 * AplasmaM1 \quad 13$$

$$\frac{d(AM2)}{dt} = fracl2 * AliverM2 - kurineM2 * AM2 \quad 14$$

$$\frac{d(AM3)}{dt} = RAML3 - kurineM3 * AM3 \quad 15$$

$$\frac{d(AM4)}{dt} = fracl4 * RAML4 + RAMG4 - kurineM4 * AM4 \tag{16}$$

$$\frac{d(AurineM2)}{dt} = kurineM2 * AM2 \tag{17}$$

$$\frac{d(AurineM3)}{dt} = kurineM3 * AM3 \tag{18}$$

$$\frac{d(AurineM4)}{dt} = kurineM4 * AM4 \tag{19}$$

$$RAMG1 = vmaxgutM1 * cgut * \frac{fu}{cgut * fu + kmgutM1} \tag{20}$$

$$RAMG1\_cyt = vmaxgut\_cytM1 * cgut * \frac{fu}{cgut * fu + kmgut\_cytM1} \tag{21}$$

$$RAMG2 = vmaxgutM2 * cgutM1 * \frac{fumi}{cgutM1 * fumi + kmgutM2} \tag{22}$$

$$RAMG3 = vmaxgutM3 * cgutM1 * \frac{fumi}{cgutM1 * fumi + kmgutM3} \tag{23}$$

$$RAMG4 = vmaxgutM4 * \frac{cgutM2}{cgutM2 + kmgutM4} \tag{24}$$

$$RAMG5 = vmaxgutM5 * cgutM1 * \frac{fumi}{cgutM1 * fumi + kmgutM5} \tag{25}$$

$$RAML1 = vmaxliverM1 * cliver * \frac{fu}{cliver * fu + kmliverM1} \tag{26}$$

$$RAML1\_cyt = vmaxliver\_cytM1 * cliver * \frac{fu}{cliver * fu + kmliver\_cytM1} \tag{27}$$

$$RAML2 = vmaxliverM2 * cliverM1 * \frac{fumi}{cliverM1 * fumi + kmliverM2} \tag{28}$$

$$RAML3 = vmaxliverM3 * cliverM1 * \frac{fumi}{cliverM1 * fumi + kmliverM3} \tag{29}$$

$$RAML4 = vmaxliverM4 * cliverM2 * \frac{cliverM2}{cliverM2 + kmliverM4} \tag{30}$$

$$RAML5 = vmaxliverM5 * cliverM1 * \frac{fumi}{cliverM1 * fumi + kmliverM5} \tag{31}$$

where,

$RAMG1$ is metabolism of DEHP into MERHP in microsomal fraction of gut;

$RAMG1\_cyt$ is metabolism of DEHP into MEHP in cytosol fraction of gut;

$RAMG2$ is metabolism of MEHP into MEHP-OH in microsomal fraction of gut;

$RAMG3$ is metabolism of MEHP into 5-carboxy MEPP;

$RAMG4$ is metabolism of MEHP-OH into 5-oxo MEPP;

$RAMG5$ is metabolism of MEHP into phthalic acid;

$RAML1$ is metabolism of DEHP into MEHP in mmicrosomal fraction of liver;

$RAML2$ is metabolism of MEHP into MEHP-OH;

$RAML3$ is metabolism of MEHp into 5-carboxy MEPP;

$RAML4$ is metabolism of MEHP-OH into 5-oxo MEPP;

$RAML5$ is metabolism of MEHP into phthalic acid;

$vmaxgutM1$ is the maximum metabolic rate of reaction in gut microsomes (DEHP to MEHP);

$vmaxgut\_cytM1$ is the maximum metabolic rate of reaction in gut cytosol (DEHP to MEHP);

$kmgutM1$ is the concentration at which half maximum reaction occur (microsomal fraction);

$kmgut\_cytM1$ is the concentration at which half maximum reaction occur (cytosol freaction);

$vmaxgutM2$ is the maximum metabolic rate of reaction for MEHP to MEHP-OH;

$kmgutM2$ is the concentration at which half maximum reaction occur (MEHP to MEHP-OH);

$vmaxgutM3$ is the maximum metabolic rate of reaction for MEHP to 5-carboxy MEPP;

$kmgutM3$ is the concentration at which half maximum reaction occur (MEHP to 5-carboxy MEPP);

$vmaxgutM4$ is the maximum metabolic rate of reaction for MEHP-OH to 5-oxo MEPP;

$kmgutM4$ is the concentration at which half maximum reaction occur (MEHP-OH to 5-oxo MEPP);

$vmaxgutM5$ is the maximum metabolic rate of reaction for MEHP to phthalic acid;

$kmgutM5$ is the concentration at which half maximum reaction occur (MEHP to phthalic acid);

$vmaxliverM1$ is the maximum metabolic rate of reaction in liver microsomes (DEHP to MEHP);

$vmaxliver\_cytM1$ is the maximum metabolic rate of reaction in liver cytosol (DEHP to MEHP);

$kmliverM1$ is the concentration at which half maximum reaction occur (microsomal fraction);

$kmliver\_cytM1$ is the concentration at which half maximum reaction occur (cytosol freaction);

$vmaxliverM2$ is the maximum metabolic rate of reaction for MEHP to MEHP-OH;

$kmliverM2$ is the concentration at which half maximum reaction occur (MEHP to MEHP-OH);

$vmaxliverM3$ is the maximum metabolic rate of reaction for MEHP to 5-carboxy MEPP;

$kmliverM3$ is the concentration at which half maximum reaction occur (MEHP to 5-carboxy MEPP);

$vmaxliverM4$ is the maximum metabolic rate of reaction for MEHP-OH to 5-oxo MEPP;

$kmliverM4$ is the concentration at which half maximum reaction occur (MEHP-OH to 5-oxo MEPP);

$vmaxliverM5$ is the maximum metabolic rate of reaction for MEHP to phthalic acid;

$kmliverM5$ is the concentration at which half maximum reaction occur (MEHP to phthalic acid);

$\frac{fumi}{fum}$ is the fractional unbound for the MEHP in plasma and microsomes are assume to be the same;

$kurine$ is the urine elimination rate constant.

*$M1$, $M2$, $M3$ and $M4$ are corresponds to MEHP, 5-OH MEHP, 5-carboxy MEPP, 5-oxo MEPP respectively.
